# Complete Genome Sequences of Three Historically Important, Spatiotemporally Distinct, and Genetically Divergent Strains of Zika Virus: MR-766, P6-740, and PRVABC-59

**DOI:** 10.1128/genomeA.00800-16

**Published:** 2016-08-18

**Authors:** Sang-Im Yun, Byung-Hak Song, Jordan C. Frank, Justin G. Julander, Irina A. Polejaeva, Christopher J. Davies, Kenneth L. White, Young-Min Lee

**Affiliations:** Department of Animal, Dairy, and Veterinary Sciences, College of Agriculture and Applied Sciences, Utah State University, Logan, Utah, USA

## Abstract

Here, we report the 10,807-nucleotide-long consensus RNA genome sequences of three spatiotemporally distinct and genetically divergent Zika virus strains, with the functionality of their genomic sequences substantiated by reverse genetics: MR-766 (African lineage, Uganda, 1947), P6-740 (Asian lineage, Malaysia, 1966), and PRVABC-59 (Asian lineage-derived American strain, Puerto Rico, 2015).

## GENOME ANNOUNCEMENT

Zika virus (ZIKV) is a positive-stranded RNA virus of the family *Flaviviridae*, genus *Flavivirus* (1). ZIKV is spread among humans primarily through the bite of infected *Aedes* species mosquitoes (2), but it can also be transmitted from an infected mother to her child during pregnancy (3–7) or by sexual contact (8, 9) or blood transfusion (10). In nature, ZIKV is probably maintained in a sylvatic cycle involving wild primates and arboreal mosquitoes (11–16). Although ZIKV infection generally causes a mild and self-limiting illness in infected individuals (17, 18), it has recently been associated with a growing number of severe neurological disorders, including Guillain-Barré syndrome in adults and microcephaly in newborns (3–5, 19–21). Historically, ZIKV spread from equatorial Africa and Asia (22–33) to the Pacific Islands (34–40), and most recently, to Latin America (41–46); it is now a pandemic in progress (47–50).

Over the past half century, a significant number of ZIKV isolates have been obtained sporadically from three continents (Africa, Asia, and America); however, little is known about the genetic variation among these geographically and temporally distinct ZIKVs because of the limited number of fully sequenced ZIKV genomes (27, 51–61). Here, we have determined the complete genome sequences of three historically important spatiotemporally distinct ZIKVs (Table 1): (i) MR-766, the first ZIKV isolated in Uganda from a sentinel rhesus monkey in 1947 (33); (ii) P6-740, the first non-African strain isolated in Malaysia from a pool of *Aedes aegypti* mosquitoes in 1966 (23); and (iii) PRVABC-59, the current American epidemic strain isolated in Puerto Rico from a human patient in 2015 (62). The consensus nucleotide sequence for each of the three genomic RNAs was determined by direct sequencing of three overlapping cDNA amplicons (nucleotides [nt] 1 to 4530, nt 2339 to 7386, and nt 5626 to 10611 [numbered based on LC002520]) that cover the entire viral genome except the 5′ and 3′ termini; 5′- and 3′-rapid amplification of cDNA ends was then performed to obtain the missing terminal sequences (nt 1 to 163 and nt 10053 to 10807), according to our established protocols (63, 64).

**TABLE 1:** Summary of the complete genome sequences of three spatiotemporally distinct genetically divergent ZIKVs with the accession numbers deposited at the GenBank sequence database

Strain	Country of isolation	Collection date	Host	Complete genome size (nt)	5′ NCR^*a*^ (nt)	ORF^*b*^ (nt)	3′ NCR (nt)	Genetic lineage	GenBank accession no.
MR-766^*c*^	Uganda	April 1947	Monkey (*Macaca mulatta*)	10,807	106	10,272	429	African	KX377335
P6-740^*d*^	Malaysia	July 1966	Mosquito (*Aedes aegypti*)	10,807	107	10,272	428	Asian	KX377336
PRVABC-59^*e*^	Puerto Rico	December 2015	Human (*Homo sapiens*)	10,807	107	10,272	428	Asian	KX377337

a NCR, noncoding region.

b ORF, open reading frame.

c The genome of MR-766 has been fully sequenced in this study and by three other independent groups (accession numbers AY632535, LC002520, and KU955594), and their nucleotide sequences are not identical, most likely because of variations in the cultivation history of the virus.

d The partial coding sequence of P6-740 has been sequenced previously (accession no. HQ234499).

e The genome of PRVABC-59 has been sequenced previously by two research groups (accession numbers KU501215 and KX087101), but both lack the 5′- and 3′-terminal sequences.

In all three ZIKV strains, the genomic RNA is 10,807 nt in length, with a single 10,272-nt open reading frame (ORF) flanked by a 106- or 107-nt 5′ noncoding region (NCR) and a 428- or 429-nt 3′ NCR (Table 1). The ORF encodes a 3,423-amino acid (aa) polyprotein predicted to be cleaved into 10 proteins: 122-aa C, 168-aa prM, 504-aa E, 352-aa NS1, 226-aa NS2A, 130-aa NS2B, 617-aa NS3, 150-aa NS4A, 251-aa NS4B, and 903-aa NS5. The functionality of all three genomic sequences was validated by generating their full-length infectious cDNA clones (our unpublished data). A phylogenetic analysis using the nucleotide sequences of the 29 available ZIKV genomes (15 complete and 14 near-complete) revealed two major genetic lineages: African, including MR-766; and Asian, including both P6-740 and PRVABC-59, with PRVABC-59 derived from an ancestor of the Asian lineage, in agreement with recent reports (6, 7, 38, 43, 62, 65, 66). Our findings provide a foundation for comparative functional genomics studies of ZIKV biology.

### Accession number(s).

The accession numbers deposited at the GenBank sequence database are listed in Table 1.
